# Recovery of high value‐added protein from enzyme‐assisted aqueous extraction (EAE) of soybeans by dead‐end ultrafiltration

**DOI:** 10.1002/fsn3.936

**Published:** 2019-01-24

**Authors:** Qiaozhi Zhang, Yang Li, Zhongjiang Wang, Baokun Qi, Xiaonan Sui, Lianzhou Jiang

**Affiliations:** ^1^ College of Food Science Northeast Agricultural University Harbin China; ^2^ National Research Center of Soybean Engineering and Technology Harbin China

**Keywords:** enzyme‐assisted aqueous extraction, functional property, nutrition value, protein rejection coefficient, skim fraction, ultrafiltration

## Abstract

The skim fraction (SF) obtained from enzyme‐assisted aqueous extraction (EAE) of soybeans is a by‐product with high protein content of up to 60.67%. As such, it is of great interest to develop an efficient method to recover protein from this fraction. In this study, the potential of dead‐end ultrafiltration (UF) in recovering skim protein extracted with different proteases was evaluated. Two polyethersulfone (PES) membranes with molecular weight cutoffs (MWCO) of 3 kDa and 5 kDa were utilized. Results revealed that the membrane with the MWCO of 5 kDa exhibited better filtration efficiency, since higher permeate flux values and lower impurity rejections were observed. Compared with Flavourzyme and Protex 7L, Alcalase 2.4L and Protex 6L exhibited stronger hydrolyzing ability, resulting in higher filtration fluxes but lower protein rejection coefficients. The recovered protein showed comparable amino acid profile to SPC, while with significantly reduced levels of trypsin inhibitors and phytate (*p *<* *0.05), indicating high quality of the recovered protein. Overall, UF can be applicable to recover high value‐added protein from EAE of soybeans and remove undesired components from the resulting protein products.

## 1 INTRODUCTION

Solvent extraction using hexane is the most commonly used method to extract oil from oil‐bearing seeds. However, the use of organic solvent leads to substantial safety and environmental issues, including risks of fire and explosion, health hazards to the human body, and contamination to the environment (Yusoff, Gordon, & Niranjan, 2015). The increasing concerns about the use of this volatile organic solvent resulted in hexane oil extraction, an energy‐intensive and operation‐complex process to overcome these problems (Campbell et al., 2011). Therefore, it is of great interest among researchers to develop a safe and ecological‐friendly method for this extraction process.

As a promising alternative to oil extraction using hexane, enzyme‐assisted aqueous extraction (EAE) has received significant attention recently. The use of enzymes in an aqueous medium allows for oil separation through the hydrolysis of protein and pseudomembranes surrounding the oil bodies (Campbell & Glatz, 2009). During EAE of soybeans, three distinct fractions are formed: a cream with emulsified oil, a protein and sugar‐rich skim, and a fiber‐rich residual (Campbell et al., 2011). The cream fraction is usually subjected to de‐emulsification to further improve the oil extraction yield (Li et al., 2014). The skim fraction (SF) is an aqueous phase that contains a small amount of emulsified oil and soluble saccharides and a significant amount of soybean protein (de Moura et al., 2013). It was estimated that almost 27 L of SF was produced for every liter of soybean oil extracted (Yao, Wang, & Wang, 2011). Therefore, efficient protein recovery from SF is a crucial and challenging step that impedes the commercial adoption of EAE process. Conventional methods for high‐purity soybean protein extraction are mainly based on their low solubility at average isoelectric point (pH 4.5) (Alibhai, Mondor, Moresoli, Ippersiel, & Lamarche, 2006). However, since the solubility of hydrolyzed protein was significantly enhanced at acidic pH, isoelectric precipitation only resulted in a total protein recovery of 30% in extracting skim protein from EAE of soybeans (Campbell & Glatz, 2010).

Pressure‐driven membrane processes, particularly ultrafiltration (UF), are separation techniques that are currently utilized in biotechnology, food processing, and pharmaceutical industry (Daufin et al., 2001; Rana & Matsuura, 2010; Rana, Matsuura, Kassim, & Ismail, 2015). Being regarded as an efficient, non‐destructive, and environmental‐friendly technology, UF has been successfully applied to fractionate, purify, or concentrate valuable components from various complex process streams (Rana, Matsuura, & Sourirajan, 2014). Recent investigations have shown that UF is effective in recovering proteins (protein hydrolysates) from soy processing effluents (Cassini, Tessaro, Marczak, & Pertile, 2010), dairy by‐products (Das, Sarkar, Sarkar, Bhattacharjee, & Bhattacharjee, 2016), and marine‐source wastewaters (Benhabiles et al., 2013), among others. However, to our knowledge, only two studies have previously utilized UF to recover skim protein from EAE of soybeans (Campbell & Glatz, 2010; de Moura, Campbell, de Almeida, Glatz, & Johnson, 2011), and only Protex 6L was applied for enzymatic hydrolysis. However, information on the performance of UF to recover skim protein extracted with different proteases is still unknown. Additionally, impact of EAE process on protein quality and functionality needs to be explored, to further identify applications of the protein products. Therefore, the aims of the present study were to investigate the efficiency of UF to recover skim protein from SF under different EAE treatments. Four commonly reported microbial proteases in EAE of soybeans (Flavourzyme, Alcalase 2.4L, Protex 7L, and Protex 6L) were utilized, and a stirring‐assisted dead‐end UF module was applied. Moreover, the impact of different extraction processes on the nutritional value and functional properties of the recovered protein was studied.

## 2 MATERIALS AND METHODS

### 2.1 Materials

Full‐fat soybean flakes were kindly provided by the Lanshan Group Corporation (Liaocheng, China). The full‐fat soybean flakes contained 21.5% oil, 42.5% protein, 32.8% carbohydrate, 3.2% ash, and 11.5% moisture (on dry weight basis). Flavourzyme (an enzyme cocktail of endopeptidases and exopeptidases) and Alcalase 2.4L (an endoproteinase from *Bacillus licheniformis*) were purchased from Novozymes (Beijing, China). Protex 7L (a neutral metalloendopeptidase from *Bacillus amyloliquefaciens*) and Protex 6L (an alkaline serine endopeptidase from *B. licheniformis*) were purchased from Genencor International (Rochester, NY, USA). Glucose, fructose, galactose, sucrose, raffinose, and stachyose standards as well as benzoyl‐DL‐arginine‐p‐nitroanalide hydrochloride (BAPA) were purchased from Sigma‐Aldrich (St. Louis, MO, USA). Molecular weight standards containing thyroglobulin (670 kDa), bovine globulin (158 kDa), chicken ovalbumin (44 kDa), equine myoglobin (17 kDa), and vitamin B12 (1.35 kDa) were purchased from Bio‐Rad Laboratories (Richmond, CA, USA). All other reagents were of analytical grade unless otherwise specified.

### 2.2 Collection of skim fraction (SF) from enzyme‐assisted aqueous extraction (EAE) of soybeans

Prior to EAE, the moisture content of the soybean flakes was adjusted to 14.5% by mixing the flakes with water in a mixer (model T20, K‐TRON AG, Hillenbrand, France). The moistured soybean flakes were then extruded in a twin‐screw extruder (25 mm screw diameter, Evolum 25, Clextral, Firminy, France) at a consistent screw rotation speed of 300 rpm. The extruder barrel (800 mm length) was composed of six heating modules operated at 30‐50‐70‐80‐80‐80°C. The extrudates were collected and cooled to room temperature for subsequent extraction.

Enzyme‐assisted aqueous extraction of soybeans was conducted according to the parameters in our previous work (Li et al., 2014). Briefly, the soybean extrudates were ground, sieved, and blended with deionized (DI) water at a solid‐to‐liquid ratio of 1:6 (w/v). Four commonly used microbial proteases, Flavourzyme, Alcalase 2.4L, Protex 7L, and Protex 6L, were added into the slurries at a dosage of 2.0% (v/w, on dry weight of ground soybean extrudate), respectively. The pH and temperature were maintained within the optimal range of each enzyme throughout the process according to the suppliers. At the end of reaction, the enzyme was inactivated by heating the slurry in boiling water for 15 min. After centrifugation at 8,000 *g* for 20 min at 4°C, the SF was collected, lyophilized, and referred to as SF_F_, SF_A_, SF_P7_, and SF_P6_ for Flavourzyme, Alcalase 2.4L, Protex 7L, and Protex 6L, respectively.

### 2.3 Sodium dodecyl sulfate polyacrylamide gel electrophoresis (SDS‐PAGE)

Sodium dodecyl sulfate polyacrylamide gel electrophoresis was performed to characterize the proteins in SF extracted with different proteases. The samples were diluted with 2× Laemmli sample buffer followed by heating in boiling water for 5 min. After that, aliquots of 10 μl of each sample were loaded into a 4%–15% gradient gel (35 μg protein/well) and run at 120 V for 2 hr. The gel was then stained with Coomassie Brilliant Blue R250 and destained with 10% acetic acid and 5% ethanol in DI water. Gel image was captured using a Gel Doc^™^ EZ imager (Bio‐Rad Laboratories Inc., Hercules, CA, USA).

### 2.4 Recovery of protein from SF by dead‐end ultrafiltration (UF)

**Figure 1:**
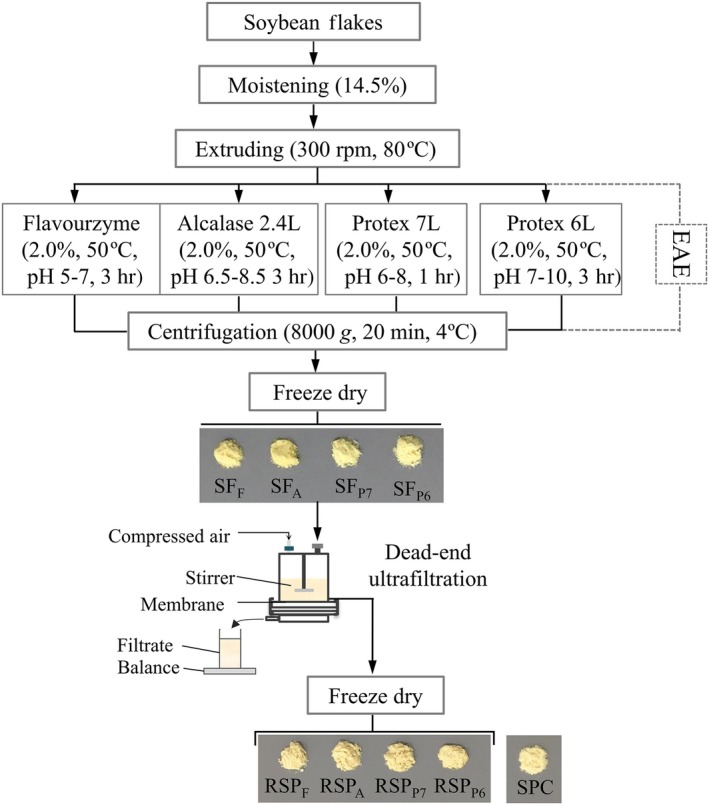
Process flow diagram of EAE of soybeans using different proteases followed by skim protein recovery by dead‐end UF. EAE, enzyme‐assisted aqueous extraction; UF, ultrafiltration

Recovery of skim protein was performed using a dead‐end UF module (Amicon 8200; Millipore, Billerica, USA), with a maximal volume of 200 ml and an effective membrane area of 2.87 × 10^−3^ m^2^. The schematic diagram of the UF module as well as the process flow is shown in Figure 1. SF was diluted with DI water to obtain a concentration of 50 mg/ml. One hundred fifty milliliter of SF solution was injected into the cell, and 30 ml of retentate was obtained. Two polyethersulfone (PES) membranes (Sepro membranes, Oceanside, USA) with molecular weight cutoffs (MWCO) of 3 and 5 kDa were used. For each experiment, The UF process was held at room temperature by applying a transmembrane pressure of 3 bar. In order to reduce the concentration polarization effect, stirring of the feed solution was conducted using a magnetic stirrer fixed over the membrane surface and rotating at a constant rate of 450 rpm. The permeate flux was monitored throughout the process by recording the volume of permeate with a graduated cylinder and calculated as (Yang et al., 2014):

(1) $$J\;(L/m^{2}\text{hr})=\frac{1}{A}\frac{dV_{p}}{dT}$$

where A is the effective membrane area (m^2^), $V_{p}$ is the total volume of permeate (L), and T is the filtration time (hr).

After UF, the permeate and corresponding retentate were collected for subsequent analysis. The apparent rejection coefficients of solutes (RC) were defined as (Zhu et al., 2016):

(2) $$R_{\mathrm{app}}\;\left(\%\right)=\left(1-\frac{C_{p}}{C_{f}}\right)\;\times \;100$$

where $C_{p}$ and $C_{f}$ are the concentrations (mg/ml) of solutes (protein, sucrose, or stachyose) in the permeate and feed solution, respectively. The retentate was lyophilized and referred to as RSP_P7_, RSP_A_, RSP_F,_ and RSP_P6_ for SP_P7_, SP_A_, SP_F,_ and SP_P6_, respectively. A new PES membrane was used for each UF experiment to avoid a loss of permeability due to membrane fouling.

### 2.5 Compositional analysis

The soluble protein concentration was quantified using the Lowry method (Lowry, Rosebrough, Farr, & Randall, 1951). Total oil content was determined using the Mojonnier method (AOAC Method 922.06), and total protein content was determined using the Kjeldahl method (AOCS Method Ba 4d‐90) (AOCS 2006). The soluble mono‐ and oligosaccharides were analyzed by HPLC (Waters e2695; Milford, MA, USA) equipped with a refractive index detector. 0.3 ml of SF or recovered skim protein (RSP) solution (1 mg/ml, in DI water) was mixed with 0.7 ml of acetonitrile to precipitate the proteins. The mixture was then centrifuged at 10,000 *g* for 10 min, and 10 μl supernatant was injected into a BEH Amide Column (150 × 4.6 mm, 3.5 μm, Waters, Hertfordshire, UK). An isocratic elution system using 75% acetonitrile and 0.2% trimethylamine in DI water was conducted. The flow rate was 0.6 ml/min, and the column temperature was set at 45°C. The peaks were identified by comparing their retention times with external standards and quantified by external calibration curves.

### 2.6 Molecular weight (MW) distribution analysis

Molecular weight distribution of SF and corresponding RSP was analyzed by gel permeation chromatography. An ÄKTA purifier system (GE Healthcare), equipped with a Superdex 75 10/300 GL column (GE Healthcare), was used. The samples were dissolved in DI water (1 mg/ml) and filtered through a 0.45‐μm filter. Five hundred microliter filtrate was then loaded into the column and eluted with 0.05 M sodium phosphate buffer containing 0.15 M NaCl (pH 6.8) (de Moura et al., 2013). The flow rate was 0.6 ml/min, and the detection wavelength was set at 214 nm. Data collection was performed using the Unicorn Software (Version 5.01; GE Healthcare, Uppsala, Sweden). The MW range of each sample was calculated based on the calibration curve of the MW standards.

### 2.7 Amino acid (AA) composition analysis

Recovered skim protein from different EAE processes was analyzed for their AA composition. 25 mg sample was hydrolyzed in 10 ml of 6 M HCl under vacuum at 110°C for 24 hr. The acid was then evaporated, and the residue was dissolved in 67 mM trisodium citrate‐HCl buffer (pH 2.2). The mixture was filtered through a 0.22‐μm filter, and 20 μl filtrate was injected into an automatic AA analyzer (Hitachi L‐8800, Tokyo, Japan). Cation‐exchange separation of the AAs was performed according to the method of Mišurcová et al. (2014).

For sulfur‐containing AAs (Met and Cys) determination, the samples were oxidized with performic acid prior to HCl hydrolysis. Tryptophan was degraded during the hydrolysis and as such its content could not be detected. Each AA was quantified with respect to their corresponding external standards and expressed as g/100 g sample.

### 2.8 Determination of trypsin inhibitor activity (TIA) and phytate content

Two antinutritional factors, the trypsin inhibitors and phytic acid (phytate), in RSP from different EAE treatments, were determined. The TIA was measured according to the method of Hamerstrand, Black, and Glover (1981) using benzoyl‐DL‐arginine‐p‐nitroanalide hydrochloride (BAPA) as a substrate, and the results were expressed as mg trypsin inhibited/g sample. The phytate content was determined using a spectrophotometric assay as described by Vaintraub and Lapteva (1988), with sodium phytate as a standard.

### 2.9 Functional property analysis

The protein solubility (PS) of RSP was measured according to McIntyre, O'Sullivan, and O'Riordan (2016) and calculated using the following equation:

(3) $$\text{PS}\;\left(\%\right)=\frac{m_{\sup}}{m_{\mathrm{ini}}}\;\times \;100$$

where $m_{\sup}$ and $m_{\mathrm{ini}}$ are the protein contents (g/100 g) in the supernatant and initial solution, respectively.

The emulsifying activity index (EAI) and stability index (ESI) of RSP were determined following the method of Liu, Shim, Poth, and Reaney (2016). Briefly, freshly prepared RSP solution (2% in DI water, w/v) was mixed with soybean oil at a ratio of 3:1 (v/v), followed by homogenization using a homogenizer (FA25 Model, Fluko Equipment Co. Ltd., Shanghai, China) at 44,000 *g* for 1 min to prepare the emulsion. After 15 min, the emulsion was diluted 1,000‐fold with 0.1% SDS (w/v) and the absorbance at 500 nm was recorded. The EAI and ESI were calculated using the following equations:

(4) $$\text{EAI}\;\left(m^{2}/g\right)=\frac{2\;\times \;2.303\;\times \;A_{0}\;\times \;V}{10,000\;\times \;\varphi \;\times \;c}$$

(5) $$\text{ESI}\;\left(\text{min}\right)=\frac{A_{0}}{A_{0}-A_{15}}\;\times \;t$$

where $A_{0}$ and $A_{15}$ are the emulsion absorbance at 0 and 15 min, respectively; V is the dilution factor (1,000); φ is the oil volume fraction (0.25); c is the initial protein concentration (g/ml); and t is the time interval (15 min).

The foaming capacity (FC) and foaming stability (FS) of RSP were measured according to Dombrowski, Dechau, and Kulozik (2016), by examining the change in foam volume and liquid volume over time. Fifty milliliter of RSP solution (0.5% in DI water, w/v) was poured into a graduated glass column (30 × 2.2 cm). Nitrogen gas at a flow rate of 150 ml/min was passed into the RSP solution for 30 s to generate foam (foam formation). FC and FS were calculated using the following equations:

(6) $$\text{FC}\;\left(\%\right)=\frac{V_{f,t=0}}{V_{l,i}-V_{l,t=0}}\;\times \;100$$

(7) $$\text{FS}\;\left(\%\right)=\frac{V_{f,t=30}}{V_{f,t=0}}\;\times \;100$$

where $V_{f,t=0}$ and $V_{f,t=30}$ are the foam volumes (ml) at 0 and 30 min after gas sparging, respectively; $V_{l,i}$ terms the loading volume of the protein solution (50 ml); and $V_{l,t=0}$ is the remaining liquid volume (ml) just after foam formation.

### 2.10 Statistical analysis

All experiments were conducted at least in triplicate, and the results were expressed as mean ± standard deviation. One‐way analysis of variance (ANOVA) with Duncan's multiple range test (*p *<* *0.05) was conducted using SPSS 22.0 s0oftware (SPSS Inc., Chicago, IL, USA).

## 3 RESULTS AND DISCUSSION

### 3.1 Chemical composition of SF

**Figure 2:**
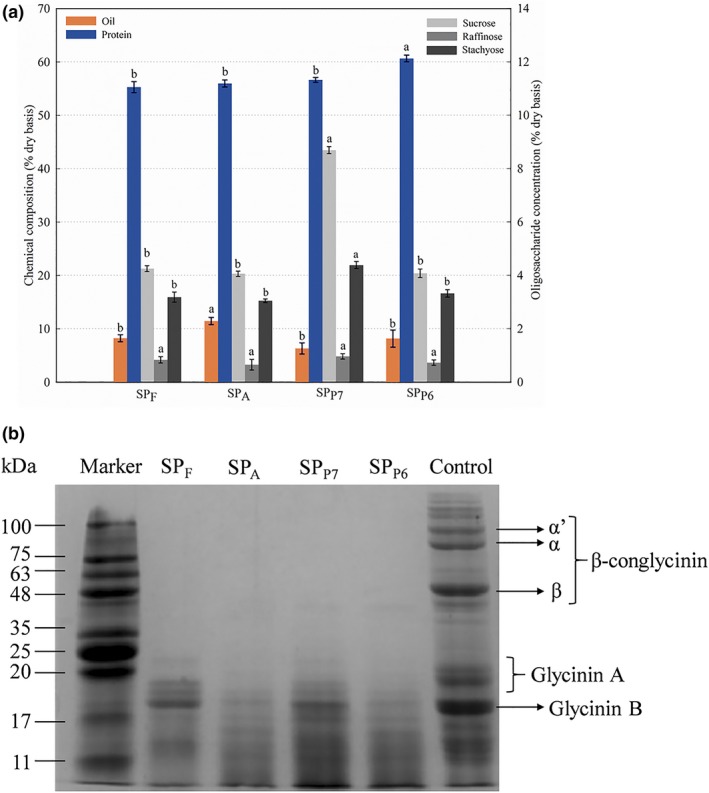
Characterization of SF extracted from different EAE treatments. (A) Chemical composition and oligosaccharide distribution; (B) SDS‐PAGE profile. Control, EAE treatment without enzyme addition. Bars with different letters in the same group indicate statistical difference (*p* < 0.05, Duncan's test). EAE, enzyme‐assisted aqueous extraction; SF, skim fraction; UF, ultrafiltration

The chemical composition of SF extracted with different proteases is shown in Figure 2A. As seen, SF contains a small amount of oil, ranging from 6.33% to 11.48% (on dry weight basis). Alcalase 2.4L tends to have a greater ability to “release” oil from soybean cytoplasm into SF, resulting in a significantly higher oil content in SF_A_ (*p *<* *0.05). A large proportion of SF is soybean protein, varying from 55.31% to 60.67% (on dry weight basis). Protex 6L exhibited the most potent ability to extract soybean protein into SF, as indicated by the highest level of protein in SF_P6_ (*p *<* *0.05). Other components in SF are soluble carbohydrates and ashes. The major saccharides in SF were sucrose, followed by stachyose, and raffinose, with the contents of 4.06%–8.69%, 3.05%–4.38%, and 0.65%–0.96% (on dry weight basis), respectively. This is in accordance with previous results (de Moura et al., 2013), showing that the main carbohydrates in SF from EAE of soybeans were stachyose and sucrose. Among the four extractions, non‐significant differences in sugar profiles were observed except for a significantly higher content of sucrose and stachyose in SF_F_ (*p *<* *0.05).

### 3.2 SDS‐PAGE profile of SF

Sodium dodecyl sulfate polyacrylamide gel electrophoresis was performed to characterize the protein profiles in SF from different EAE treatments. As shown in Figure 2B, compared to the control treatment without enzymes, proteolytic hydrolysis reduced all the subunits of the two main soybean storage proteins (glycinin and β‐conglycinin). On the whole, SF showed smaller bands with MW <48 kDa, of which the proteins with MW <20 kDa accounted for the largest proportion. Similarly, de Moura et al. (2008) reported that 71% of the skim protein extracted with Protex 6L were below the MW of 20 kDa. Furthermore, it is interesting to notice that there are hydrolyzed products (bands of MW 35–48 kDa) of the β subunit of β‐conglycinin in SF_F_ and SF_P6_. As previously noted, β‐conglycinin was more resistant to enzymatic degradation than glycinin and therefore considered as a potential food allergen (Krishnan, Kim, Jang, & Kerley, 2009). However, as for SF_P7_ and SF_A_, all the subunits of β‐conglycinin were completely absent, indicating the higher hydrolyzing abilities of Protex 7L and Alcalase 2.4L toward β‐conglycinin. This result suggested that different EAE processes generated skim proteins with different compositions and therefore may impact the quality and functionality of the resulting products.

### 3.3 Skim protein recovery using ultrafiltration (UF)

**Figure 3:**
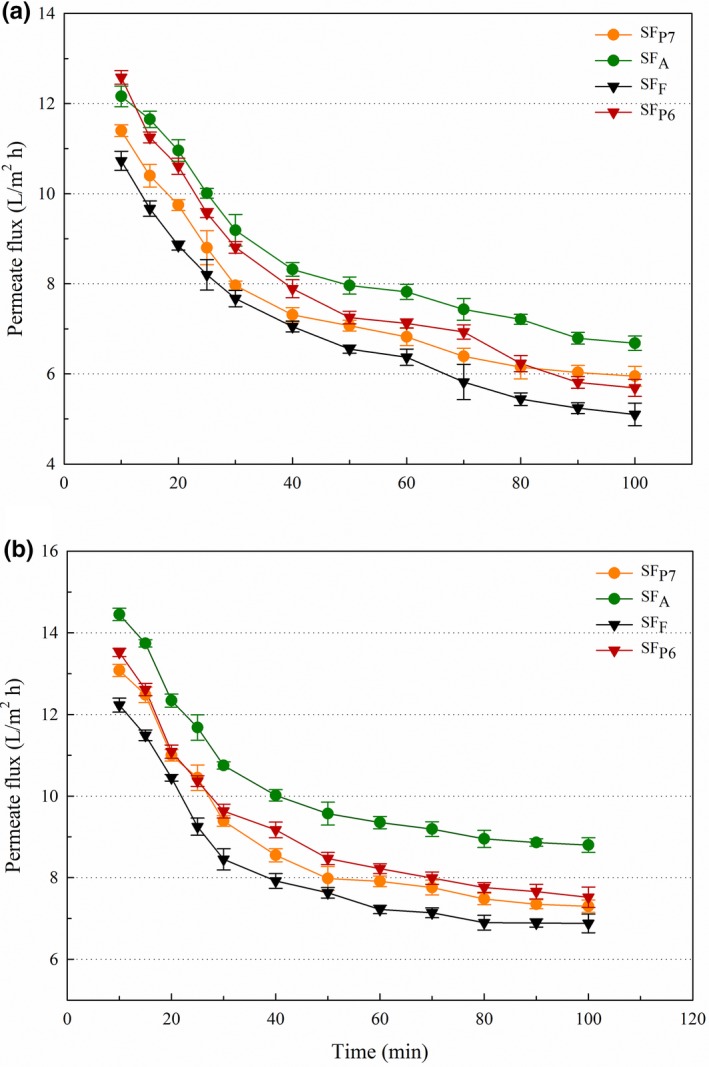
Impact of membrane MWCO on permeate flux values during UF. (A) Membranes with the MWCO of 3 kDa; (B) Membranes with the MWCO of 5 kDa. MWCO, molecular weight cut‐offs; UF, ultrafiltration

In order to evaluate the impact of membrane pore size on UF performance and retentate quality, two PES membranes with MWCO of 3 kDa and 5 kDa were utilized. Figure 3A shows the variation of permeate flux during UF for both membranes. As seen, the permeate flux values were apparently dependent on the MWCO of membranes, where the values for the 5‐kDa membrane were noticeably higher than those for the 3‐kDa membrane. As referred in the introduction, Campbell and Glatz (2010) also applied UF to recover skim protein from EAE of soybeans and the permeate flux was reported to be within 4–10 L/m^2^/hr with a 3‐kDa membrane. It should be noted that the permeate flux values we obtained here (4.73–12.58 and 8.64–14.45 L/m^2^/hr for 3‐kDa membrane and 5‐kDa membrane, respectively) are slightly higher than those reported in the previous study. Explanation can be found in the differences in raw material, SF composition (due to different EAE processes), UF equipment and parameters, among others. Overall, SF_A_ and SF_P6_ exhibited higher permeate flux values than SF_P7_ and SF_F_, and the lowest values were expectedly observed in SF_F_ with the 3‐kDa membrane. During the filtration process, the permeate flux decreased with increasing filtration time. A rapid initial flux decline, followed by a pseudo‐steady stage, was observed in all samples, albeit at different rates. It was indicated that a steep decrease in the initial flux during UF was associated with membrane fouling (Zhu et al., 2016). On the beginning of filtration, accumulation of macromolecular solutes may form a concentration polarization layer on the membrane surface, resulting in a steeply reduced flux. Beyond the layer buildup period, the filtration resistance reached a stationary state and the flux remained almost unchanged (Grenier, Meireles, Aimar, & Carvin, 2008).

Table 1 presents the retention coefficients of protein, sucrose, and stachyose (two main saccharides in SF) for each SF using the studied membranes. A UF membrane with high retention of protein but low rejection of saccharides is desired. As seen, the 3‐kDa membrane expectedly retained more protein than the 5‐kDa membrane, but significant differences were observed only for the UF process of SF_A_ and SF_P6_ (*p *<* *0.05). Among the four proteases used, filtration of the SF extracted with Flavourzyme showed the highest *R*_app_ values for protein. This could be explained by the less degree of protein hydrolysis in SP_F_, resulting in larger size difference between the protein fraction and other solutes. While for sucrose and stachyose, they have much lower MWs (180.16 kDa and 666.59 kDa, respectively) than the MWCO of both membranes, and their transmission through the pores freely was expected. However, as seen in Table 1, filtration with the 3‐kDa membrane exhibited significantly higher *R*_app_ values for stachyose when compared to the 5‐kDa membrane (*p *<* *0.05). This is probably due to the increasing concentration polarization and fouling during UF using the 3‐kDa membrane, which consequently leads to partial sugar retention in the retentates. Therefore, considering the high permeate flux values, slightly reduced retention of protein, and low rejection of saccharides, the membrane with the MWCO of 5 kDa was preferred to recover skim protein from SF and it was then selected for the following studies.

**Table 1:** Impact of membrane MWCO on solutes rejection in SF

*R*_app_ (%)	MWCO of the membrane							
	3 kDa				5 kDa			
	SF_P7_	SF_A_	SF_F_	SF_P6_	SF_P7_	SF_A_	SF_F_	SF_P6_
Protein (%)	86.93 ± 0.34^b^	83.13 ± 0.57^d^	91.42 ± 0.81^a^	85.32 ± 0.78^c^	86.51 ± 0.21^b^	80.18 ± 0.39^e^	90.61 ± 0.33^a^	82.00 ± 0.69^de^
Sucrose (%)	8.46 ± 0.09^a^	8.70 ± 0.10^a^	8.48 ± 0.15^a^	8.76 ± 0.07^a^	8.44 ± 0.16^a^	8. 66 ± 0.13^a^	8.35 ± 0.09^a^	8.68 ± 0.05^a^
Stachyose (%)	13.63 ± 0.12^c^	15.56 ± 0.09^a^	11.85 ± 0.08^ef^	14.79 ± 0.15^b^	11.52 ± 0.17^f^	12.41 ± 0.08^d^	8.62 ± 0.10^g^	12.16 ± 0.13^de^

Notes

Mean values within the same row followed by the same superscript letters are not significantly different (*p* > 0.05, Duncan's test).

MWCO: molecular weight cutoffs; SF: skim fraction.

The protein content of RSP was then evaluated, and the results are shown in Table 2. As seen, RSP contained 72.60%–82.36% protein (on dry weight basis), with the highest purity observed in RSP_F_ (*p* < 0.05). As previously reported by Campbell and Glatz (2010), a 70% protein content of the RSP extracted with Protex 6L was obtained after membrane filtration. The higher protein content in our original SF can be a possible explanation, indicating that different EAE treatments might result in SF with various chemical profiles and thereby impact the quality of the protein retentate. It is also worth noting that the protein content of RSP was slightly higher than that of commercial SPC (~70%), but lower than that of commercial SPI (~90%) (Alibhai et al., 2006). This high purity of RSP further implied its potential applications as soybean protein products, such as protein supplements or food ingredients.

**Table 2:** Amino acid composition and anti‐nutritional factors in RSP

	RSP_P7_	RSP_A_	RSP_F_	RSP_P6_	SPC
Essential amino acids (g/100 g sample)					
Lys	6.09 ± 0.04	6.01 ± 0.09	5.92 ± 0.06	6.14 ± 0.05	6.18 ± 0.06
Leu	7.56 ± 0.05	7.51 ± 0.08	7.54 ± 0.02	7.58 ± 0.07	7.57 ± 0.08
Ile	3.15 ± 0.03	3.08 ± 0.03	3.09 ± 0.08	3.16 ± 0.04	3.14 ± 0.06
Val	3.90 ± 0.06	4.10 ± 0.10	4.14 ± 0.06	3.47 ± 0.08	3.41 ± 0.04
Thr	3.92 ± 0.08	3.94 ± 0.05	3.81 ± 0.08	3.68 ± 0.04	3.53 ± 0.03
His	2.40 ± 0.08	2.41 ± 0.07	2.49 ± 0.05	2.63 ± 0.08	2.33 ± 0.05
Phe	5.24 ± 0.09	5.19 ± 0.05	5.29 ± 0.05	5.32 ± 0.05	5.49 ± 0.05
Met	1.45 ± 0.05	1.46 ± 0.02	1.52 ± 0.03	1.66 ± 0.03	1.92 ± 0.02
Non‐essential amino acids (g/100 g sample)					
Ala	3.80 ± 0.03	3.95 ± 0.08	3.63 ± 0.05	3.89 ± 0.09	3.41 ± 0.07
Arg	7.46 ± 0.07	7.49 ± 0.05	7.69 ± 0.04	7.63 ± 0.06	7.41 ± 0.05
Asp	12.66 ± 0.04	12.62 ± 0.08	13.08 ± 0.03	12.80 ± 0.05	12.54 ± 0.10
Cys	1.88 ± 0.02	1.76 ± 0.02	1.86 ± 0.02	2.00 ± 0.04	1.80 ± 0.03
Glu	21.60 ± 0.15	21.12 ± 0.11	21.48 ± 0.13	20.59 ± 0.15	22.13 ± 0.15
Gly	4.33 ± 0.08	4.44 ± 0.05	3.97 ± 0.08	4.49 ± 0.08	4.28 ± 0.05
Pro	5.46 ± 0.02	5.78 ± 0.05	5.75 ± 0.08	5.42 ± 0.01	6.01 ± 0.03
Ser	5.55 ± 0.05	5.44 ± 0.03	5.43 ± 0.05	5.70 ± 0.03	5.31 ± 0.04
Tyr	3.57 ± 0.03	3.71 ± 0.04	3.22 ± 0.06	3.84 ± 0.03	3.55 ± 0.05
ΣEAAs	33.70 ± 0.10^a^	33.69 ± 0.19^a^	33.79 ± 0.15^a^	33.65 ± 0.12^a^	33.57 ± 0.15^a^
Protein content (%, dwb)	76.93 ± 1.78^ab^	72.60 ± 3.23^b^	82.36 ± 0.56^a^	73.94 ± 2.45^ab^	–
TIA (mg trypsin inhibited/g)	3.72 ± 0.20^b^	3.81 ± 0.16^b^	3.97 ± 0.08^b^	3.98 ± 0.09^b^	9.12 ± 0.10^a^
Phytate (mg/g)	6.14 ± 0.09^b^	3.51 ± 0.08^d^	6.12 ± 0.14^b^	4.06 ± 0.06^c^	12.35 ± 0.09^a^

Notes

Mean values within the same row followed by the same superscript letters are not significantly different (*p* > 0.05, Duncan's test).

RSP: recovered skim protein; TIA: trypsin inhibitor activity.

### 3.4 Molecular weight (MW) distribution

**Figure 4:**
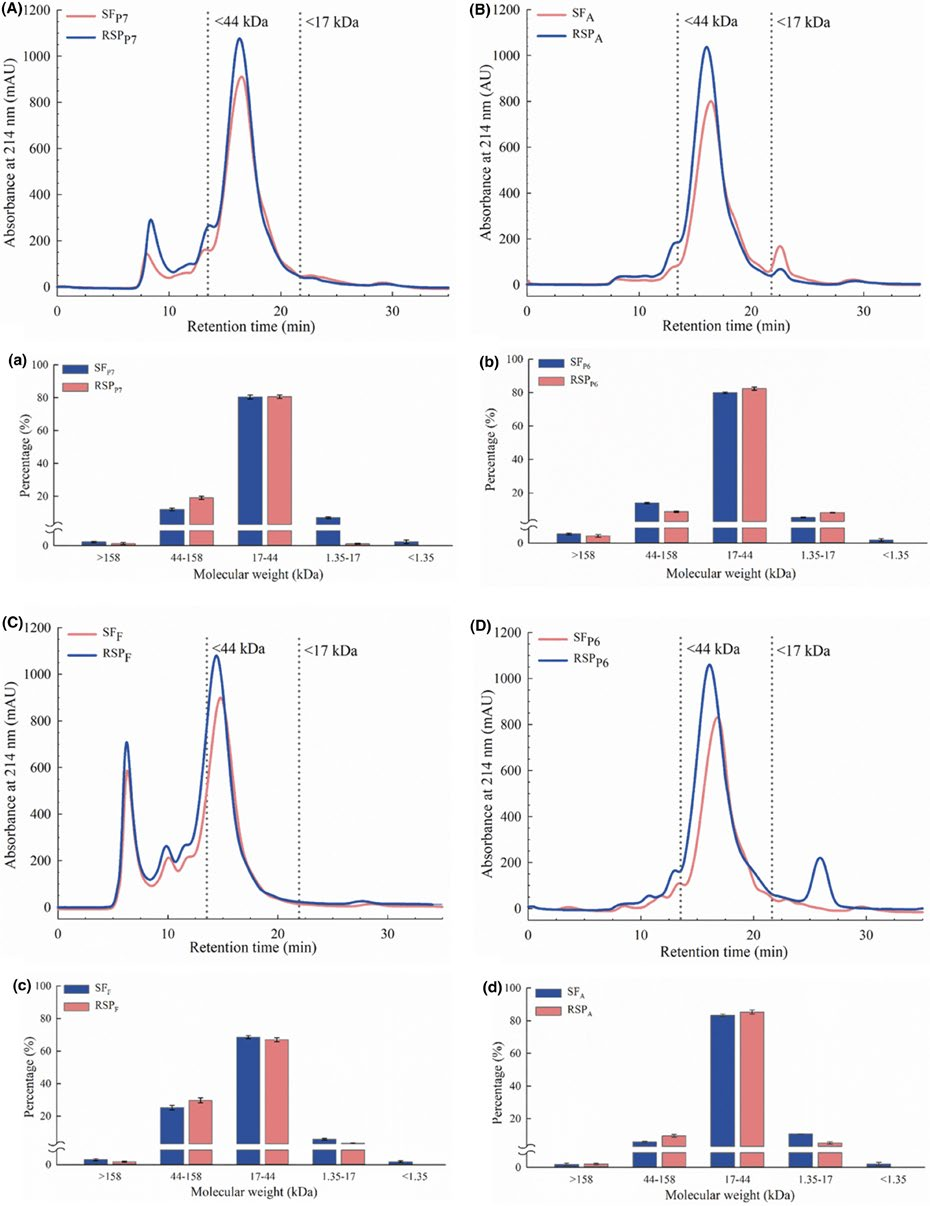
Impact of UF on protein composition of SF and corresponding RSP from different EAE treatments. (A–D) Size‐exclusion chromatograms; (a–d) Molecular weight distributions. EAE, enzyme‐assisted aqueous extraction; RSP, recovered skim protein; SF, skim fraction; UF, ultrafiltration

In order to evaluate the impact of UF on the composition of SF, MW distribution of each SP and corresponding RSP was determined and the results are shown in Figure 4. Similar to the results of SDS‐PAGE, extraction with Protex 7L and Flavourzyme yielded proteins with larger MW, as reflected by the peaks eluted before the first dashed line (Figure 4A,C). However, those peaks with larger MW were almost diminished in Figure 4B,D, indicating the higher hydrolyzing abilities of Alcalase 2.4L and Protex 6L. Moreover, a minor peak eluted after the second dash line was observed in SF_A_, implying that Alcalase 2.4L was able to further degrade the soybean protein into low MW peptides.

The MW distribution of each sample was then calculated and shown as the bar charts in Figure 4a–d. Overall, proteins with MW >17 kDa accounted for approximately 90% in all SF, of which the fractions within MW of 17–44 kDa accounted for the largest proportion. Alcalase 2.4L showed the strongest hydrolyzing ability, as indicated by the highest percentage of the proteins within MW of 17–44 kDa (83.27 ± 0.75%). Comparably, Campbell and Glatz (2010) reported that the skim protein recovered from EAE of soybeans (extracted with Protex 6L) was predominantly composed of the fractions with MW >13 kDa. After filtration, the MW profiles of RSP exhibited various changes. Specifically, the 44–158 kDa fraction was increased in RSP_F_, RSP_A,_ and RSP_P7_ following UF, and the 1.35–17 fraction was decreased in corresponding RSP accordingly. Non‐significant changes in contents of the 17–44 kDa fraction were observed among the four treatments before and after UF. However, since the membranes we used cannot retain short‐chain peptides, the fraction with MW <1.35 kDa was completely diminished in all resulting retentates.

### 3.5 Amino acid (AA) composition and anti‐nutritional factors of RSP

Amino acid composition is an important factor to evaluate the nutritional value of protein and protein hydrolysates. The AA profiles of RSP from different enzyme treatments are shown in Table 2 and compared with that of SPC (extracted from the same seeds). As seen, RSP was rich in Glu, Asp, and Ile, but were relatively deficient in Met and Cys. This is in accordance with the results of Zarkadas et al. (2007), who analyzed the AA composition of 14 soybean cultivars and found that they were high in glutamic acid but limited in methionine and cysteine. Overall, both of the contents of essential AAs and non‐essential AAs in RSP were similar to those in SPC, indicating the high quality of the recovered protein. Similar to our results, Latif, Pfannstiel, Makkar, and Becker (2013) reported that the AA composition of SF from EAE of peanuts was close to that of the untreated ground seed. Meanwhile, although some AA differs, on the whole, non‐significant differences were observed among different EAE processes. de Moura et al. (2008) also reported comparable AA compositions of the skim protein extracted under different enzyme species and dosages (Protex 6L and Protex 7L). Overall, RSP contained all the necessary AAs, which further indicated the gently operational conditions during EAE and UF process.

There are a number of anti‐nutritional compounds in soybeans that can negatively impact their nutritional quality (Liener, 1994). In this study, two anti‐nutritional factors, the trypsin inhibitors and phytate, were determined in RSP from different EAE processes. The trypsin inhibitors belong to the family of serine protease inhibitors, and function through complexing with trypsin and chymotrypsin, resulting in interference of the protein digestive process (Vagadia, Vanga, & Raghavan, 2017). As shown in Table 2, compared with SPC, RSP showed significantly lower levels of TIA, varying from 3.72 to 3.98 mg trypsin inhibited/g sample (*p *<* *0.05). It is known that trypsin inhibitors are heat‐labile and could be thermally inactivated. Therefore, the reduced levels of TIA in RSP could be probably due to the hydrothermal treatment during extrusion. However, since the extrusion temperature we used was relatively low (80°C), the trypsin inhibitors were not completely denatured, and there still remained partial TIA in the resulting RSP. Additionally, employment of UF may further reduce the TIA levels since the 1.35–17 kDa fractions in RSP were depleted (Figure 4), which is near the MW of Bowman–Birk (6–10 kDa) inhibitor, one major trypsin inhibitor in soybeans (Liener, 1994).

Phytate is a phosphorous compound that can impede the absorption of certain minerals in the gut, such as zinc and iron (Lazarte, Carlsson, Almgren, Sandberg, & Granfeldt, 2015). As previously evidenced, there is a strong interaction between phytate and soybean protein, making it a difficult task to remove phytate during protein extraction (Yang et al., 2014), and as a result, SPC contains 12.35 mg/g sample phytate (Table 1). However, it appears that enzymatic treatment during EAE weakens the protein–phytate interaction through proteolysis, resulting in partial removal of phytate during the filtration. This is confirmed by the significantly lower levels of phytate content in RSP, as compared to that in SPC (*p *<* *0.05). Similarly, Latif et al. (2013) reported that the phytate content in SF from EAE of peanuts was significantly lower than those in the Soxhlet extracted residue and cold‐pressed residue. Among the four EAE treatments, RSP_A_ showed the lowest phytate content (3.51 mg/g sample, *p *<* *0.05), which could be due to the higher hydrolyzing ability of Alcalase 2.4L.

### 3.6 Functional properties of RSP

**Figure 5:**
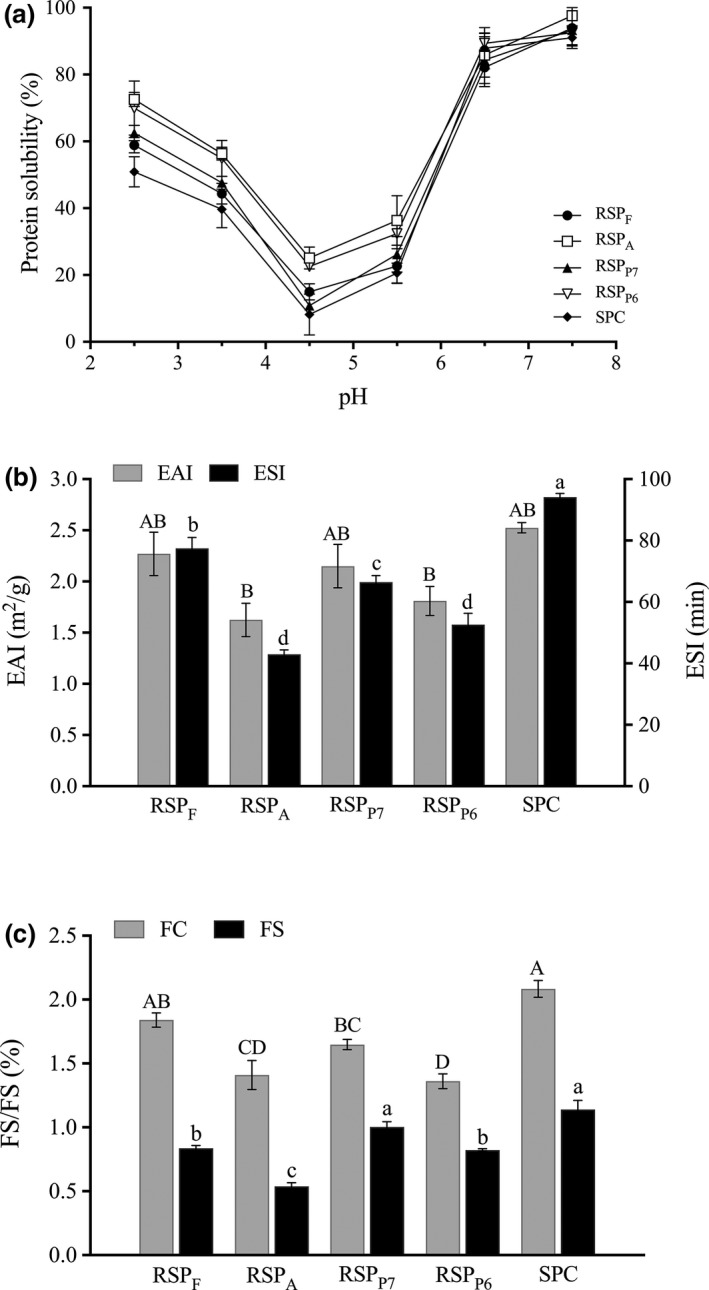
Functional properties of RSP recovered from different EAE treatments. (A) Protein solubility; (B) Emulsifying property; (C) Foaming property. Bars with different letters in the same group indicate statistical difference (*p* < 0.05, Duncan's test). EAE, enzyme‐assisted aqueous extraction; EAI, emulsifying activity index; ESI, emulsifying stability index; FC, foaming capacity; FS, foaming stability; RSP, recovered skim protein

Processing treatments, such as heating, pH adjustment, and enzymatic hydrolysis, may impact the physicochemical properties of protein and thereby alter their functionalities (Foegeding & Davis, 2011). In this study, to further evaluate the effect of EAE treatments on functional properties of RSP, some certain properties, including protein solubility (PS), emulsifying and foaming properties, were determined. The improvement of PS following enzymatic treatment is known to be the most notable impact on protein functionalities (Zhao, Liu, Zhao, Ren, & Yang, 2011). Figure 5A shows the PS‐pH profiles of RSP extracted with different proteases and compared with that of SPC. As seen, both RSP and SPC exhibited typical U‐shaped curves with the lowest PS at pH 4.5, which is the isoelectric point of soybean protein. Enzymatic treatments improved the PS of RSP in most cases, especially at acidic pH. This PS‐enhancing effect of PS after proteolysis can be attributed to the reduced MW as well as the increase in ionizable and carboxyl groups. Among the four proteases, Alcalase 2.4L and Protex 6L exhibited better PS‐improving abilities, achieving the PS values of 97.61% and 92.52% at pH 7.5, respectively. The solubility of SF from EAE of soybeans using Protex 6L was previously reported by de Almeida, de Moura Bell, and Johnson (2014), but with much lower values (approx. 53% at pH 7.0), which is possibly due to the higher protein purities we obtained in RSP in this study.

Emulsifying activity index (EAI) and stability index (ESI) refer to the maximum interfacial area per gram protein required to stabilize the solution, and its capacity to remain unchanged over time under given conditions (Lam & Nickerson, 2013). Soybean proteins are known as effective emulsifiers, and therefore, they are widely used in food formulations (Tang, 2017). However, as shown in Figure 5B, compared to SPC, EAE treatments significantly (*p *<* *0.05) reduced the EAI of RSP_A_ and RSP_P6_ and ESI of all RSP, respectively. Similar trends were observed in foaming properties (Figure 5C), where the FC and FS of RSP were decreased by 11.70%–34.68% and 11.97%–52.69%, respectively, as compared to the SPC. Limited enzymatic modification was shown to improve the emulsifying and foaming properties of plant proteins (Avramenko, Low, & Nickerson, 2013; Tang, 2017). However, when the hydrolyzing process was not well controlled, the proteins were cleaved into peptides with reduced ability to form viscoelastic films at the interface, thereby causing coalescence of oil droplets and destabilization of foams (Ghribi et al., 2015). Hence, the reduction in interfacial properties of RSP in this study could be due to the excessive protein hydrolysis during EAE process. Changing extraction parameters would be beneficial to control the process and obtain skim proteins with preferable properties, for instance, reducing enzyme dosage or hydrolyzing time. However, the oil extraction yield would be altered accordingly, as well as the stability of the oil‐rich emulsion. As such, optimization of the whole process would be necessary to further fulfill simultaneous extraction of the desired products.

## 4 CONCLUSIONS

Application of UF in this study demonstrated its possibility to recover high‐quality protein from the liquid fraction of EAE of soybeans. Results showed that the membrane with the MWCO of 5 kDa exhibited better filtration performance, as it had higher permeate fluxes and lower impurity rejections. An overall protein rejection of 80.18%–90.61% was obtained using the 5‐kDa membrane, with high purities of 72.60%–82.36% obtained in the retentates. The recovered proteins contained all the necessary AAs, while consisting reduced levels of soybean anti‐nutritional factors. Enzymatic hydrolysis improved protein solubility of the recovered proteins but decreased their emulsifying and foaming properties. These results suggest that UF can be an effective way to recover high value‐added skim protein from EAE of soybeans. However, since undesirable flux reduction was observed during the filtration, more effective anti‐fouling and membrane cleaning methods are still expected.

## CONFLICT OF INTEREST

The authors declare that they have no conflicts of interest.

## ETHICAL STATEMENT

This study did not include any human or animal experiments.
